# Pathogenesis of Painful Diabetic Neuropathy

**DOI:** 10.1155/2014/412041

**Published:** 2014-05-06

**Authors:** Amir Aslam, Jaipaul Singh, Satyan Rajbhandari

**Affiliations:** ^1^Department of Diabetes, Lancashire Teaching Hospital NHS Trust, Chorley and South Ribble District General Hospital, Preston Road, Chorley PR7 1PP, UK; ^2^School of Pharmacy and Biomedical Sciences and School of Forensic and Investigative Sciences, University of Central Lancashire, Preston, Lancashire PR1 2HE, UK

## Abstract

The prevalence of diabetes is rising globally and, as a result, its associated complications are also rising. Painful diabetic neuropathy (PDN) is a well-known complication of diabetes and the most common cause of all neuropathic pain. About one-third of all diabetes patients suffer from PDN. It has a huge effect on a person's daily life, both physically and mentally. Despite huge advances in diabetes and neurology, the exact mechanism of pain causation in PDN is still not clear. The origin of pain could be in the peripheral nerves of the central nervous system. In this review, we discuss various possible mechanisms of the pathogenesis of pain in PDN. We discuss the role of hyperglycaemia in altering the physiology of peripheral nerves. We also describe central mechanisms of pain.

## 1. Introduction

Diabetes affects 382 million people wordlwide and its prevalence is expected to increase to 592 million by the year 2035 [1]. Diabetic neuropathy, a well-known, long-term complication of diabetes, can affect almost half of the diabetic population [2] and is associated with higher morbidity and mortality [3]. Diabetic neuropathy encompasses a variety of clinical or subclinical presentations. Painful diabetic neuropathy (PDN) is a common type of diabetic neuropathy and the most common cause of neuropathic pain [4]. The reported prevalence of PDN varied from 11% in Rochester, Minnesota, USA [5], to 53.7% in the Middle East [6]. One UK study published in 2011 reported that the prevalence of PDN was 21.5% in type 2 diabetes patients and 13.4% in type 1 diabetes patients, resulting in an overall prevalence of 21% [7]. In the large, prospective EURODIAB study in 16 European countries, almost one-quarter of type 1 patients developed new onset painful diabetic neuropathy over a seven-year period [8]. A prospective study in Finland followed newly diagnosed diabetes patients between the ages of 45 and 64 years for 10 years. It found a 6% prevalence at the time of diagnosis of diabetes and a 26.4% prevalence at the 10-year follow-up [9]. In a large UK-based community diabetic population, Abbot et al. [7] observed that increasing age was directly related to painful symptoms of neuropathy. Most studies found no significant difference in gender; however, Abbot et al. [7] reported a slightly higher prevalence of painful symptoms of neuropathy in females (38%) than males (31%). The same study also found a higher prevalence of painful symptoms in South Asians (38%) compared to Europeans (32%).

Painful diabetic neuropathy (PDN) symptoms exhibit a symmetrical “stocking and gloves” distribution and are often associated with nocturnal exacerbation. It can be presented from a mild pins and needle sensation to stabbing, burning, unremitting, or even unpleasant electric shock sensation. There can be allodynia in the form of cutaneous hypersensitivity leading to acute distress on contact with an external stimulus, such as clothing [10]. The pain is often worse at night and often disturbs sleep, causing tiredness during the day. Some patients present with distressing allodynia and severe pain in the legs. This may be so painful that it prevents them from performing their daily activities, thereby impacting their employment and social life. The constant, unremitting pain and withdrawal from social life often result in depression [11]. In extreme cases, patients lose their appetite and experience significant weight loss, which is reported in the literature as “diabetic neuropathic cachexia” [10].

## 2. Physiology of Pain

Pain is the body's perception of actual or potential damage to the nerve or tissue by noxious stimuli. The sensory afferent nerves carry sensations from the skin, joints, and viscera via large and small fibres. Large fibres, such as A-alpha, are responsible for limb proprioception and A-beta fibres carry sensations of limb proprioception, pressure, and vibration. Large A-delta myelinated fibres and small C unmyelinated fibres are mainly responsible for carrying nociceptive sensations. Superficial pain is often a sharp or pricking sensation and is transmitted by A-delta fibres. A deep-seated, burning, itching, aching type of pain is often accompanied with hyperalgesia and allodynia and is transmitted via slow, unmyelinated C fibres. Tissue damage results in the release of inflammatory chemicals, such as prostaglandins, bradykinins, and histamines, at the site of inflammation, which triggers the depolarization of nociceptors, thereby generating an action potential. The action potential transmits the nociceptive sensation, via the dorsal root ganglion (DRG), to the dorsal horn of the spinal cord. The release of glutamate and substance P results in the relay of nociceptive sensations to the spinothalamic tract, thalamus, and, subsequently, the cortex, where pain is interpreted and perceived [12].

Nociceptive pain is the normal response to noxious insult or injury of tissues such as skin, muscles, visceral organs, and joints. Nociceptive pain usually subsides upon the healing of the tissue injury. On the other hand, neuropathic pain arises as a direct consequence of a lesion or disease affecting the somatosensory system without any noxious stimuli. Neuropathic pain is caused by damage or pathological change and is characterised by the activation of abnormal pathways of pain at the peripheral nerves and posterior roots (peripheral neuropathic pain) or spinal cord and brain (central pain) [13]. Neuropathic pain manifestation can be focal, multifocal, or generalized depending on the involvement of peripheral or central origin and cause of the disease. A few examples of neuropathic pain are listed in Table 1.

## 3. Neuropathic Pain Generation Pathogenesis

The origin of pain in PDN is not fully understood. The abnormalities in the peripheral or central nervous system could be related to hyperglycaemia, as this is the key metabolic abnormality of diabetes. There are many other conditions that produce pain similar to that of PDN and they may also aid our understanding of the pathophysiology of PDN.

### 3.1. Ectopic Electrical Impulses

Chronic hyperglycemic damage to the nerves can cause regeneration of nerve sprouts, called neuromas, at the stump. The sprouting of the new nerves in all directions causes collateral damage of otherwise undamaged nerves and expands the sensitized area [14]. Hyperexcitability in the neuroma generates ectopic impulses that affect neighbouring intact afferents and the cell bodies of the DRG. It leads to spontaneous, exaggerated, abnormal hyperexcited responses, along with increased sensitivity to a given stimulus [15]. This phenomenon is called peripheral sensitization. Electrical impulses from the axon of small fibres at the dorsal horn of the spinal cord are increased and, hence, it alters the “gate” (described below) and causes the release of substance P and glutamate. This causes a relay of the impulses to the ascending track, which is perceived as pain [16].

### 3.2. Change in Glucose Flux and Pain

Treatment of induced acute neuropathy due to rapid glycemic control in the first month of the initiation of insulin or oral hypoglycemic agents has been reported in the literature as “insulin neuritis.” In 1992, Boulton [17] first described the observation that acute painful neuropathy might follow a sudden change in glycaemia control, suggesting that blood glucose flux could precipitate pain. This observation was experimentally tested in rats by Kihara et al. in 1994 [18]. In their study, they infused insulin under nonhypoglycemic conditions and evaluated its effect on endoneurial oxygen tension, nerve blood flow, and the oxyhaemoglobin dissociation curve of peripheral nerves in normal and diabetic rats. Their results showed that insulin administration caused a reduction in nerve nutritive blood flow and an increase in arteriovenous shunt flow. When the arteriovenous shunts were obliterated by the infusion of 5-hydroxytryptamine, endoneurial oxygen reverted to normal. Sudden changes in glycaemia may induce relative hypoxia in nerve fibres, which contributes to the generation of impulses, thereby indicating that it is the combination of structural and functional changes in peripheral nerves that cause the pain.

**Figure 1:**
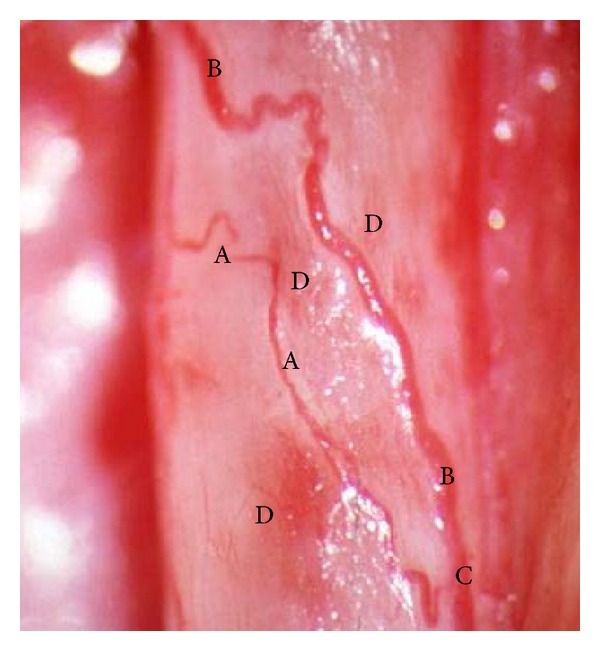
Arteriolar attenuation (A), tortuosity (B), arteriovenous shunting (C), and proliferation of newly formed vessels (D) of the vasa nervosum seen in the sural nerve of a patient with insulin neuritis (photo courtesy of Tesfaye).

In 1996, Tesfaye et al. [19] observed neurovascular changes in vivo in five human diabetic patients with insulin neuritis. These patients presented with severe sensory symptoms but clinical examination and electrophysiological tests were normal, except in one subject who had severe autonomic neuropathy. On sural nerve exposure in vivo, epineural blood vessels showed severe structural abnormalities resembling the retinopathy changes normally seen in the retina, including arteriolar attenuation, tortuosity, arteriovenous shunting, and the proliferation of newly formed vessels. They hypothesized that the structural abnormalities in epineural blood vessels, together with the formation of new vessels, caused a steal effect and, hence, resulted in hypoxia and neuropathic pain. It can now be postulated that a sudden change in glycemic control can cause flux effects that result in structural and functional changes in the epineural blood vessels of nerves, which, in turn, can lead to neuropathic pain or “insulin neuritis” [17, 19] (Figure 1). Symptoms can be mild and often go unreported but may present with severe, excruciating neuropathic pain. Symptoms usually last up to six months and respond to treatment that is usually needed for up to six months [10].

### 3.3. Role of the Dorsal Root Ganglion in Neuropathic Pain

The expression of voltage-gated sodium and calcium channels and voltage-independent potassium channels in the DRG has a significant role in the generation of nociceptive sensation and peripheral sensitization that leads to central sensitization. Hyperexcited ectopic impulses are generated by the expression of various voltage-gated sodium channels, such as Nav1.3, Nav1.7, and Nav1.8 [20]. The voltage-gated sodium channel Nav1.3 probably plays a key role in the development of neuropathic pain [21]. Amir et al. described after nerve injury, in the DRG, the fact that there is a sustained phasic discharge that results in repeated firing [22]. The voltage-dependent sodium channel alternates with a voltage-independent potassium leak to oscillate membrane potentials. When these oscillations reach the threshold amplitude, they result in the generation of ectopic impulses and, hence, lead to sustained peripheral sensitization [23]. In addition to the voltage-gated sodium channels, the expression of voltage-gated calcium channels was also found in neuropathic pain [24]; specifically subtype Cav 3.2 is highly expressed in DRG neurons and showed strong correlation with allodynia [25]. Calcium entry through voltage-gated calcium channels causes the release of substance P and glutamate, which results in the modulation of pain at the dorsal horn [26]. The upregulation of transient receptor potential expression is also found to be associated with neuropathic pain. Studies found a direct relationship between TRPV1 (transient receptor potential vanilloid 1) and neuropathic pain. A few animal studies suggest that hyperalgesia does not develop in TRPV1-deficient mice and TRPV1 antagonists reduce pain behaviour in mice [27, 28].

### 3.4. Methylglyoxal and Pain

Methylglyoxal (MG) is a reactive intracellular by-product of several metabolic pathways. However, the most important source of MG is glycolysis and hyperglycaemia [29]. Studies found that PDN patients had significantly higher concentration of plasma MG (>600 nM) compared to healthy control or diabetes patients without pain [30, 31]. MG depolarizes the sensory neuron by activating TRPV1 in the DRG [32] and also induces posttranslational modification of the voltage-gated sodium channel Nav1.8 [30]. These changes are associated with increased electrical excitability and facilitate firing of nociceptive neurons.

### 3.5. Sympathetic Modulation of Pain

Nociceptive A-delta and C fibres are normally not directly connected to sympathetic nervous system. Several experiments using *α*-adrenoreceptor agonists found that it did not activate sympathetic neurons at nociceptor fibres under normal conditions [33, 34]. It is widely accepted that the sympathetic nervous system does not activate the sensory nervous system under normal conditions.

Neuropathy causes hypersensitivity in nerves as a result of an abnormal epinephrine-mediated transmission from one axon to another. This unusual connection is called ephaptic transmission or cross-talk [35]. It was also noted that damaged nerves in the periphery also cause basket formation, called sympathetic sprouting in the DRG, which results in the release of noradrenaline [36]. Both sympathetic sprouting and ephaptic transmission release adrenaline and cause sympathetic-sensory coupling. This leads to an increase in ectopic and spontaneous firing. This unusual connection is called sympathetically maintained pain.

Several studies proved this hypothesis and showed dramatic improvement in pain relief after sympathetic blockage [37], sympathectomy [38], or temporary blockage with *α*-adrenergic antagonists with intravenous phentolamine [39].

### 3.6. Gate Control Theory

In 1965, Melzak and Wall [40] described, for the first time, the fact that nervous connections from the peripheral to central nervous system and to the brain are not a seamless transmission of information. They described the gate mechanism at the dorsal horn of the spinal cord, which inhibits or facilitates the flow of afferent impulses from peripheral nerves to the spinal cord before it evokes pain perception. The activity at the gate is primarily dependent on the transmission of impulses along small or large nerve fibres. Small nerve fibres, unmyelinated C fibres, and myelinated A-delta fibres tend to open the gate and large A-beta fibres tend to close the gate. Opening and closing of the gate depend on the number of input impulses. Thus, if nociceptive input from C- and A-delta fibres exceeds A-beta fibre input, then the gate is open and nociceptive impulses ascend to the spinal cord. On the other hand, if A-beta fibre input (touch, vibration, and pressure) exceeds that of C- and A-delta fibre input (pain), then the gate is closed; nociceptive impulses only pass through when the gate is open. The classic example of this phenomenon is the rubbing of an injured site immediately after suffering from trauma, which results in gate closure.

### 3.7. Central Sensitization

Central sensitization was first described by Woolf in 1983. Nonnoxious stimuli transmitted from the periphery with A-beta fibres (touch) were perceived as painful by patients with allodynia [41]. A-delta fibres and C fibres are innervated in laminae I-II and A-delta fibres also are innervated in lamina V of the dorsal horn. The majority of spinal cord neurons that express the substance P receptor are located in lamina I or have their cell bodies in laminae III-IV but extend their dendrites to lamina I. The pain mediation of noxious stimuli occurs by releasing substance P, mainly in lamina I of the dorsal horn. A-beta fibres are innervated deep in laminae III to V and are responsible for touch mediation [42–44]. Peripheral sensitization and sustained hyperexcited impulses at the dorsal horn cause an increase in responsiveness to noxious and nonnoxious stimulation. This was believed to be due to the structural plasticity of sprouting of A-beta fibres, which leads to “rewiring” of the dorsal horn laminae in the central nervous system (CNS) [44]. As a result, the CNS pathway, which is responsible for transmitting only nonnoxious stimuli (touch), was replaced by sprouting A-beta fibres that transmit nonnoxious impulses and release substance P in the dorsal horn, thereby mediating allodynia [45]. This hypothesis was mainly based on experiments that showed that the uptake of the cholera toxin B (CTB) subunit, which is a selective tracer for large myelinated A-fibres, terminated in lamina II [46]. The selectivity of this toxin after peripheral nerve injury is somewhat controversial. Experiments demonstrated that uptake of the CTB tracer was not selective, that CTB was found in axons of all types, including A-delta fibres and C fibres, and that the CTB tracer incorporated in C fibres that terminated in lamina II [47]. This contradicts the hypothesis of structural plasticity and A-beta fibres sprouting in lamina II. However, studies with immunostaining and electrophysiological recordings have clearly established that peripheral nerve injury causes large myelinated fibres to begin to drive nociceptive neurons in superficial lamina [48, 49]. The persistent incoming nerve impulses lead to activation of N-methyl-D-aspartate (NMDA) receptors on postsynaptic membranes in the dorsal horn of the spinal cord. This leads to the release and binding of glutamate (an excitatory neurotransmitter), which causes an influx of sodium and calcium and an efflux of potassium. This generates a larger postsynaptic action potential and augments the perception of normal stimuli, thereby resulting in allodynia [50].

### 3.8. Central Inhibition

Impulses from the brainstem nuclei descend to the spinal cord and influence the transmission of pain signals at the dorsal horn. The periaqueductal grey matter (PAG), locus coeruleus, the nucleus raphe magnus, and several bulbar nuclei of reticular formation give rise to descending modulatory pathways. These pathways dampen or enhance the pain signal. The projections from the nucleus raphe magnus to the spinal cord are the major source of serotonin in the spinal cord. Exogenous opioids imitate the endogenous opioids and induce analgesia by acting upon the PAG, reticular formation, and the spinal dorsal horn [12]. The antidepressant serotonin and norepinephrine reuptake inhibitors (SNRIs) [51] and opioids [52] have been found to be beneficial in the treatment of neuropathic pain as these medications increase the availability of these neurotransmitters and, hence, increase inhibition at the spinal cord. Psychological factors, such as fear and anxiety, can influence the inhibitory mechanism through the modulatory system. Cognitive behavioural therapies are thought to be beneficial in modulating the pain by reducing the fear and anxiety [53].

### 3.9. Thalamic Abnormalities

The nociceptive hyperexcited impulse generated within primary afferent nerves is modulated and amplified not only at the DRG-spinal cord level but also at the thalamic ventral posterolateral (VPL) level, before being relayed to the cerebral cortex. This was experimentally proved in streptozosin rat model with PDN. The experiment demonstrated hyperexcitability in thalamic VPL neurons, with increased responses to phasic brush, press, and pinch stimuli applied to peripheral receptive fields. VPL neurons from diabetic rats also displayed enhanced spontaneous activity, independent of ascending afferent impulses, and enlarged receptive fields [54]. Selvarajah et al. [55] investigated this further in humans using a magnetic resonance (MR) perfusion scan in patients with PDN. This study demonstrated increased thalamic vascularity and sluggish blood flow. Similar vascular perfusion findings were also observed at the sural nerve in patients with PDN [56]. It was suggested that increased perfusion at thalamus VPL neurons in PDN patients causes an increase in neuronal activity and, hence, further modulates pain and central sensitization.

### 3.10. Chronic Neuropathic Pain and Plasticity of Brain

Neuroplasticity or plasticity of the brain is the term used to describe the adaptive change in structure, chemical balance, and function of the brain in response to changes within the body or in the external environment. In response to chronic neuropathic pain, neuroplasticity is associated with somatosensory cortex remodelling, reorganization, and hyperexcitability in the absence of external stimuli. A study of patients with chronic neuropathic and nonneuropathic pain using functional and anatomical magnetic resonance imaging found cortical reorganization and changes in somatosensory cortex activity only in the neuropathic pain group [57]. Provoked pain and spontaneous stimuli may reverse the remodelling and reorganization at the somatosensory cortex. Studies have shown a beneficial effect of pain relief with transcranial magnetic stimulation (TMS) and transcranial direct current stimulation (tDCS), which suggests a reversal of plasticity [58, 59].

## 4. Conclusion

In summary, the exact mechanism of pain in PDN is far from being clear. The source of pain could be anywhere in the pathway from the damaged nerves to the somatosensory cortex or it could be due to a combination of pathologies. PDN is a distressing condition and, as a result, adversely affects a patient's quality of life, both physically and mentally. Despite significant advances in therapeutics, the treatment of chronic symptoms of pain in PDN is still suboptimal and challenging for clinicians [11, 60]. This may be due to a poor understanding of the pathogenesis of PDN. There is an increasing body of evidence that suggests that the central nervous system is primarily responsible for maintaining painful symptoms. In recent years, there have been significant advances in the neuroimaging of pain. Further research is needed to have a better understanding of the disease process of PDN, which will help to tackle this enormous challenge.

## Figures and Tables

**Table 1:** Examples of neuropathic pain.

Origin of pain	Structure	Example
Peripheral nervous system Central nervous system	Nerve Dorsal root Spinal cord Thalamus	Diabetic painful neuropathy Neuroma Phantom limb pain Trigeminal neuralgia Lumbosacral plexopathy Postherpetic neuralgia Brachial plexus avulsion Spinal cord injury Spinal cord infarction Multiple sclerosis Infarct Tumour Parkinson's disease
